# Dietary Fibre Intake in Type 2 and New-Onset Prediabetes/Diabetes after Acute Pancreatitis: A Nested Cross-Sectional Study

**DOI:** 10.3390/nu13041112

**Published:** 2021-03-29

**Authors:** Xinye Li, Wandia Kimita, Jaelim Cho, Juyeon Ko, Sakina H. Bharmal, Maxim S. Petrov

**Affiliations:** School of Medicine, University of Auckland, Auckland 1023, New Zealand; xli949@aucklanduni.ac.nz (X.L.); wandia.kimita@auckland.ac.nz (W.K.); chojael@gmail.com (J.C.); ju.ko@auckland.ac.nz (J.K.); s.bharmal@auckland.ac.nz (S.H.B.)

**Keywords:** dietary fibre, insoluble fibre, soluble fibre, acute pancreatitis, glycated haemoglobin, fasting plasma glucose, prediabetes, diabetes

## Abstract

The association between intake of dietary fibre and glucose metabolism has been extensively investigated in numerous metabolic disorders. However, little is known about this association in individuals after an attack of acute pancreatitis (AP). The aim was to investigate the associations between intake of dietary fibre and markers of glucose metabolism in individuals with new-onset prediabetes or diabetes after acute pancreatitis (NODAP), pre-exiting type 2 prediabetes or diabetes, and normoglycaemia after acute pancreatitis. This cross-sectional study was nested within the parent prospective longitudinal cohort study. The studied markers of glucose metabolism were fasting plasma glucose and glycated haemoglobin. Habitual intake of dietary fibre was determined using the EPIC-Norfolk food frequency questionnaire. Multivariable linear regression analyses were conducted. The study included a total of 108 individuals after AP. In the NODAP group, increased intakes of total fibre (β = −0.154, *p* = 0.006), insoluble fibre (β = −0.133, *p* = 0.01), and soluble fibre (β = −0.13, *p* = 0.02) were significantly associated with a reduction in fasting plasma glucose. Increased intakes of vegetables (β = −0.069, *p* = 0.004) and nuts (β = −0.039, *p* = 0.038) were significantly associated with a reduction in fasting plasma glucose. Increased intake of nuts (β = −0.054, *p* = 0.001) was also significantly associated with a reduction in glycated haemoglobin. None of the above associations were significant in the other study groups. Habitual intake of dietary fibre was inversely associated with fasting plasma glucose in individuals with NODAP. Individuals after an attack of AP may benefit from increasing their intake of dietary fibre (specifically, vegetables and nuts) with a view to preventing NODAP.

## 1. Introduction

Post-pancreatitis diabetes mellitus, which is a subtype of diabetes of the exocrine pancreas, is the most frequent sequela of acute pancreatitis (AP) [1,2]. Epidemiological evidence showed that individuals with a history of AP are at more than two-times higher risk of developing diabetes mellitus when compared with the general population [3,4]. The 2020 LACERTA study—the first purposely-designed prospective longitudinal cohort study with regular follow-ups in the field—showed that 43% of individuals developed new-onset prediabetes or diabetes (NODAP) within 24 months after an attack of AP [5]. In addition, evidence from a population-based study showed that individuals with post-pancreatitis diabetes mellitus are more likely to receive insulin therapy by 16.8% at five years after diabetes diagnosis when compared with individuals with Type 2 diabetes [6].

Nutrition is one of the cornerstones of early management in Type 2 diabetes. The guidelines by both the American Diabetes Association [7] and Diabetes UK [8] promulgate the use of nutrition therapy for people with Type 2 diabetes as an integral part of the management of this type of diabetes. Dietary fibre, which is a complex carbohydrate mainly derived from the structural component of the plant cell wall [9], has been studied extensively in relation to glucose metabolism in the setting of Type 2 diabetes. Evidence from previous studies suggested that dietary fibre is associated with decreased plasma glucose, reduced risk of disease, and improved disease outcomes [10,11,12,13,14,15,16]. An earlier meta-analysis of randomised controlled trials in individuals with Type 2 diabetes showed that an increase in dietary fibre intake (through food high in fibre or soluble fibre supplements for at least eight weeks) resulted in a significant decrease in fasting plasma glucose (FPG) and glycated haemoglobin (HbA1c) [11]. A later systematic review of both randomised controlled trials and observational studies in adults with prediabetes, Type 1 diabetes, or Type 2 diabetes showed that increased intake of soluble dietary fibre and wholegrain led to decreased levels of FPG, HbA1c, and insulin in randomised controlled trials (with an average duration of 6 to 12 weeks) as well as with reduced all-cause mortality in prospective observational studies (with a mean follow-up of 8.8 years) [10]. Data from randomised controlled trials also suggested that increased intake of insoluble fibre was associated with significantly reduced HbA1c and improved insulin sensitivity [12,13]. Furthermore, observational studies showed that increased intake of insoluble fibre was associated with reduced risk of Type 2 diabetes, cardiovascular disease, and cancer [14,15,16]. In addition, intake of dietary fibre from common food sources was investigated in several prospective studies, which suggested that a dietary pattern with a high consumption of fruit and vegetables [17,18,19,20], legumes [21,22], and cereals [16,23,24] was associated with reduced risk of Type 2 diabetes. While in the past it was thought that only extensive pancreatic necrosis or chronic inflammation of the pancreas (resulting in β-cell destruction) can lead to the development of new-onset diabetes, several recent studies have consistently shown that glucose derangements can also develop after an attack of mild acute pancreatitis (driven by insulin resistance) [2,25,26,27]. However, to date, dietary fibre intake has never been investigated specifically in the context of NODAP.

The aim was to investigate the associations between habitual dietary fibre intake as well as its common sources in food (i.e., fruit, vegetables, cereals, nuts) and markers of glucose metabolism (i.e., FPG and HbA1c) in individuals after an attack of AP.

## 2. Methods

### 2.1. Study Design

This was a cross-sectional study of individuals after an attack of AP as part of the ANDROMEDA project. The study was nested into the ongoing prospective longitudinal cohort study by the COSMOS group [5]. The study was approved by the Health and Disability Ethics Committee (13/STH/182).

### 2.2. Study Population

Individuals admitted to Auckland City hospital (Auckland, New Zealand) with a primary diagnosis of AP between the years of 2015 and 2019 were eligible. They participated in the study if they met the inclusion criteria: (1) had a primary diagnosis of AP based on the international guidelines, (2) lived in Auckland at the time of the study, (3) were at least 18 years old, and (4) provided informed consent.

Individuals were not eligible to participate in the study if they had: (1) definite chronic pancreatitis, (2) post-endoscopic retrograde cholangiopancreatography pancreatitis, (3) intraoperative diagnosis of pancreatitis, (4) pregnancy, (5) malignancy, (6) celiac disease, (7) cystic fibrosis, (8) type 1 or gestational diabetes, and (9) a history of steroid use.

### 2.3. Study Groups

Participants were categorised into three non-overlapping groups based on their FPG and/or HbA1c levels at the time of the study, in line with the ‘DEP criteria’ [28]. Individuals with normoglycemia after acute pancreatitis (NAP) included participants with HbA1c < 5.7% (39 mmol/mol) and/or FPG < 100 mg/dL (5.6 mmol/L) at the time of the study. Individuals who were diagnosed with Type 2 diabetes or prediabetes prior to their first attack of pancreatitis and had an HbA1c ≥ 5.7% (39 mmol/mol) and/or FPG ≥ 100 mg/dL (5.6 mmol/L) at the time of the study constituted the T2DM group. Participants who were normoglycaemic before and during attack of AP and had follow-up HbA1c ≥ 5.7% (39 mmol/mol) and/or FPG ≥ 100 mg/dL (5.6 mmol/L) after hospital discharge constituted the NODAP group. Only participants who met the above criteria at more than three months after hospitalisation for AP were included in the NODAP group, in line with the published recommendations [1,2,29].

### 2.4. Ascertainment of Dietary Intake

Habitual dietary intake over 12 months prior to the study was determined using a validated semi-quantitative EPIC-Norfolk food frequency questionnaire (FFQ). The FFQ consisted of 130 food items. Participants were asked to select the frequency of consumption of each food item in the past year with a standardised portion size. Nine categories of frequencies were presented [30]. Habitual intake of non-starch polysaccharides was used for dietary fibre analysis. Non-starch polysaccharides are defined as the carbohydrate polymers present in the plant cell wall resistant to digestion in the small intestine. These comprise cellulose, hemicellulose, pectin, β-glucan, arabinoxylan, glucomannans, gums, and mucilages [31]. The FETA (FFQ EPIC Tool for Analysis) software was used to calculate nutrient intake based on the UK food composition database, McCance and Widdowson’s ‘The Composition of Foods’ (5th edition) and associated supplements [32]. Dietary fibre intake (grams) was calculated as the amount of fibre present in 100 g of food multiplied by the portion size and multiplied by the frequency of consumption, which is in line with the published recommendations [33]. Insoluble, soluble, and total (sum of insoluble and soluble) fibre intake was determined. In addition, the intake of the most common sources of dietary fibre (vegetables, fruit, cereals, and nuts) was determined. In line with earlier studies on habitual dietary intake [34,35,36,37], FFQs were excluded if more than ten food items were left unanswered or if the total energy intake estimate derived from FFQ as a ratio of the individual’s estimated basal metabolic rate (determined based on the Harris-Benedict equation) was more than two standard deviations outside the mean of this ratio (<−0.18 or >2.54). Intake of fibre supplements or other nutritional supplements was not considered due to the limitation of the used software.

### 2.5. Laboratory Assays

All participants were required to fast for at least 8 hours before blood sample collection. Certified phlebotomists collected fresh blood samples for measuring FPG and HbA1c at LabPlus—The international accreditation New Zealand (IANZ) accredited medical laboratory at Auckland City Hospital. FPG was measured using an enzymatic colourimetric assay (©2015 F. Hoffmann-La Roche Ltd., Basel, Switzerland). HbA1c was analysed using the boronate affinity chromatography assay (©2015 Roche Products (New Zealand) Ltd., Auckland, New Zealand and Roche Diagnostics NZ Ltd., Auckland, New Zealand).

### 2.6. Covariates

Body mass index (BMI) (kg/m^2^) was determined using a stadiometer (Health o meter^®^ Professional 2013, Pelstar LLC, McCook, IL, USA) by measuring height (cm) and weight (kg). Height measurement required participants to remove shoes and head attire while, for weight measurement, participants were asked to remove shoes, jackets, belts, watches, and empty the pocket of any item before measurement. Energy intake was defined as the average daily intake of calories (kcal) from food consumption assessed using the FFQ and determined by the FETA software [30,33]. Use of anti-diabetic medications prior to the study was recorded. Aetiology of AP was categorised into biliary and non-biliary (including alcohol-related and other). Recurrence of AP was defined as one or more episodes of AP from the participant’s first hospitalisation for AP to the time of the study visit. Presence of pancreatic necrosis was determined with the use of computed tomography during hospitalisation for AP [38].

### 2.7. Statistical Analyses

Data on baseline characteristics were presented as a median and interquartile range (IQR) for continuous variables and frequencies for categorical variables. Total fibre, insoluble fibre, soluble fibre, vegetables, fruit, nuts, cereals, energy intake, HbA1c, and FPG were log-transformed because of their non-normal distribution (based on the Shapiro-Wilk test). Multivariable linear regression analyses were performed to investigate the associations between dietary fibre intake (total fibre, insoluble fibre, and soluble fibre) and markers of glucose metabolism (FPG and HbA1c). Four models were built. Model 1 was unadjusted. Model 2 was adjusted for age and sex. Model 3 was adjusted for age, sex, BMI, energy intake, and use of anti-diabetic medications. Model 4 was adjusted for age, sex, BMI, energy intake, use of anti-diabetic medications, aetiology of AP, recurrence of AP, and the presence of pancreatic necrosis. Data were presented as a β coefficient, adjusted R^2^, and *p* value. *p* values less than 0.05 were considered statistically significant in all analyses. All statistical analyses were conducted using IBM SPSS Statistics for Windows, version 26 (IBM Corp., Armonk, NY, USA) and SAS 9.4 (SAS Institute Inc., Cary, NC, USA).

## 3. Results

### 3.1. Study Characteristics

A total of 108 individuals after an attack of AP met all the eligibility criteria, of whom 36 belonged in the NODAP group, 36 belonged in the T2DM group, and 36 belonged in the NAP group. The mean time since last attack of AP was 27 months (standard error of 2 months). The frequency of prediabetes did not differ significantly between the NODAP and T2DM groups (78% vs. 67%, *p* = 0.579). Baseline characteristics of the three groups are shown in Table 1. In the overall cohort, the median (IQR) daily intake of total fibre was 12.8 (9.1–18.6) g, insoluble fibre was 9.5 (6.6–15.2) g, and soluble fibre was 3.4 (1.9–5.4) g. With regard to the sources of fibre, the median (IQR) daily intake of vegetables was 207.5 (113.2–321.3) g, of fruit was 114.2 (51.1–276.6) g, of cereals was 173.3 (115.2–281.5) g, and of nuts was 4.7 (2.1–17.1) g.

### 3.2. Fibre Intake in the Study Groups

**Figure 1:**
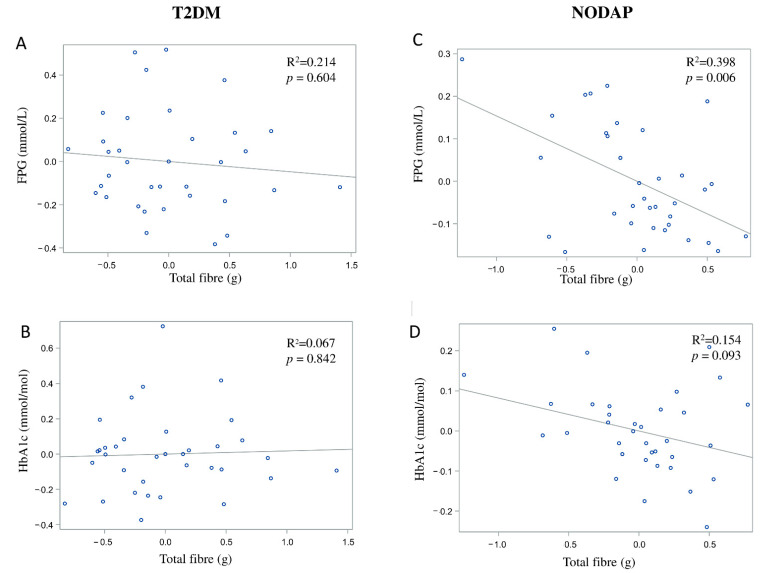
Associations between total fibre intake and markers of glucose metabolism in T2DM (**A**,**B**) and NODAP (**C**,**D**). Abbreviations: T2DM = Type 2 diabetes or prediabetes prior to acute pancreatitis. NODAP = New-onset diabetes or prediabetes after acute pancreatitis. HbA1c = glycated haemoglobin. FPG = fasting plasma glucose. R^2^ = adjusted R^2^ value (coefficient determinations). Footnotes: FPG, HbA1c, and total fibre data were log-transformed. R^2^ values were adjusted for age, sex, BMI, energy intake (log-transformed), use of anti-diabetic medications, aetiology of pancreatitis, recurrence of pancreatitis, and presence of pancreatic necrosis.

**Figure 2:**
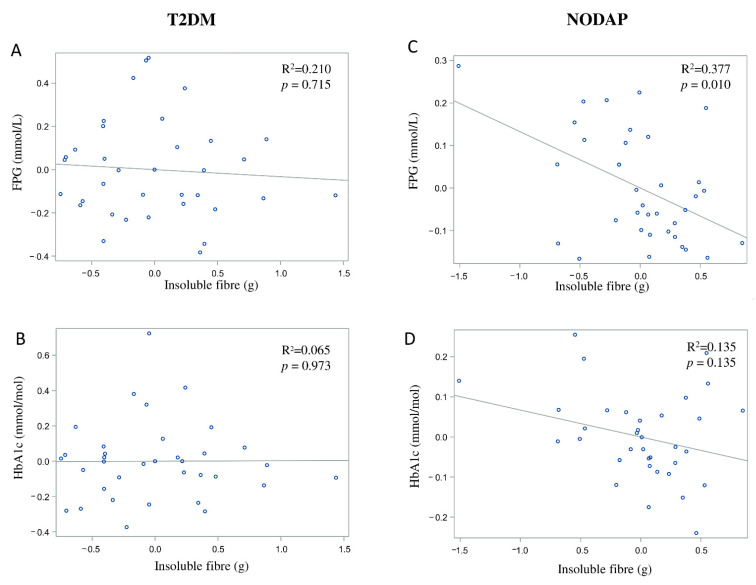
Associations between insoluble fibre intake and markers of glucose metabolism in T2DM (**A**,**B**) and NODAP (**C**,**D**). Abbreviations: T2DM = Type 2 diabetes or prediabetes prior to acute pancreatitis. NODAP = New-onset diabetes or prediabetes after acute pancreatitis. HbA1c = glycated haemoglobin. FPG = fasting plasma glucose. R^2^ = adjusted R^2^ value (coefficient determinations). Footnotes: FPG, HbA1c, and insoluble fibre were log-transformed. R^2^ values were adjusted for age, sex, BMI, energy intake (log-transformed), use of anti-diabetic medications, aetiology of pancreatitis, recurrence of pancreatitis, and presence of pancreatic necrosis.

**Figure 3:**
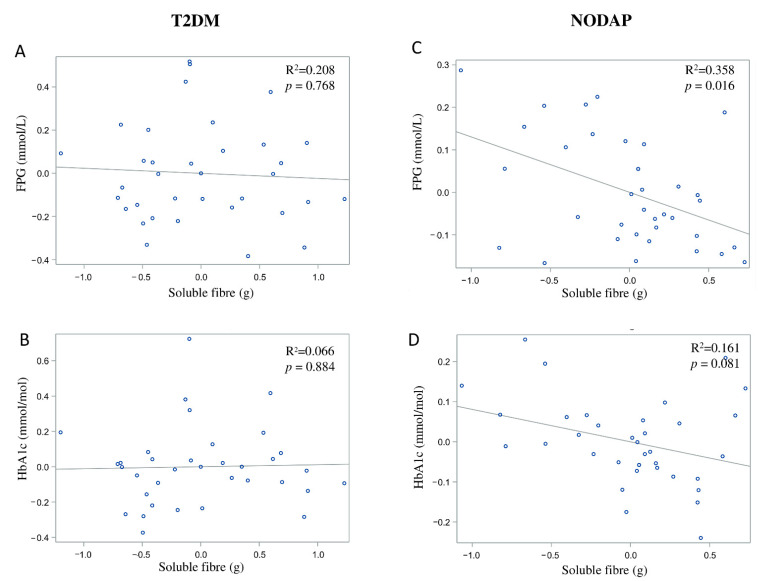
Associations between soluble fibre intake and makers of glucose metabolism between T2DM (**A**,**B**) and NODAP group (**C**,**D**). Abbreviations: T2DM = Type 2 diabetes or prediabetes prior to acute pancreatitis. NODAP = New-onset diabetes or prediabetes after acute pancreatitis. HbA1c = glycated haemoglobin. FPG = fasting plasma glucose. R^2^ = adjusted R^2^ value (coefficient determinations). Footnotes: FPG, HbA1c, and soluble fibre were log-transformed. R^2^ values were adjusted for age, sex, BMI, energy intake (log-transformed), use of anti-diabetic medications, aetiology of pancreatitis, recurrence of pancreatitis, and presence of pancreatic necrosis.

In the NODAP group, intake of total fibre, insoluble fibre, and soluble fibre was significantly inversely associated with FPG in models 3 and 4 (Table 2). Specifically, in the most adjusted model, every 1% increase in intake of total fibre, insoluble fibre, and soluble fibre, was associated with a 0.15%, a 0.13%, and a 0.13% decrease in FPG, correspondingly (Figure 1C, Figure 2C and Figure 3C). Intake of total fibre and soluble fibre was significantly inversely associated with HbA1c in model 3 (Table 3), but became insignificant in the most adjusted model (Figure 1D and Figure 3D). Intake of insoluble fibre was not significantly associated with HbA1c in any model.

In the T2DM group, neither total fibre nor insoluble fibre nor soluble fibre was significantly associated with FPG in any model (Table 2). Similarly, neither total fibre nor insoluble fibre nor soluble fibre was significantly associated with HbA1c in any model (Table 3). Associations between total fibre, insoluble fibre, soluble fibre intake, and glucose metabolism in the T2DM group are shown in Figure 1A,B, Figure 2A,B and Figure 3A,B.

In the NAP group, intake of soluble fibre was significantly inversely associated with FPG in adjusted models (Table 2). Specifically, in the most adjusted model, every 1% increase in intake of soluble fibre was associated with a 0.12% decrease in FPG. Neither intake of total fibre nor insoluble fibre was significantly associated with FPG. Although intake of total fibre and insoluble fibre was significantly directly associated with HbA1c in models 1 and 2, it became insignificant in models 3 and 4 (Table 3). Intake of soluble fibre was not significantly associated with HbA1c in any model (Table 3).

### 3.3. Dietary Sources of Fibre in the Study Groups

**Figure 4:**
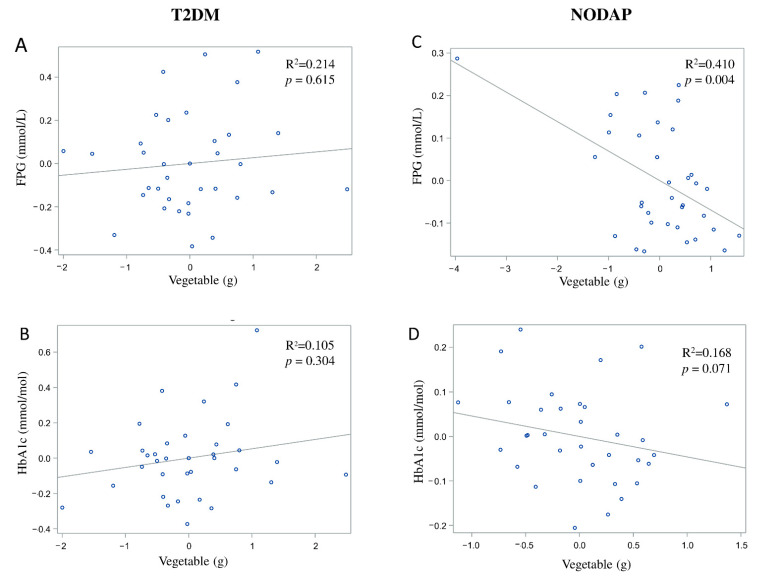
Associations between vegetables intake and markers of glucose metabolism between T2DM (**A**,**B**) and NODAP group (**C**,**D**). Abbreviations: T2DM = Type 2 diabetes or prediabetes prior to acute pancreatitis. NODAP = New-onset diabetes or prediabetes after acute pancreatitis. HbA1c = glycated haemoglobin. FPG = fasting plasma glucose. R^2^ = adjusted R^2^ value (coefficient determinations). Footnotes: FPG, HbA1c, and vegetables were log-transformed. R^2^ values were adjusted for age, sex, BMI, energy intake (log-transformed), use of anti-diabetic medications, aetiology of pancreatitis, recurrence of pancreatitis, and presence of pancreatic necrosis.

**Figure 5:**
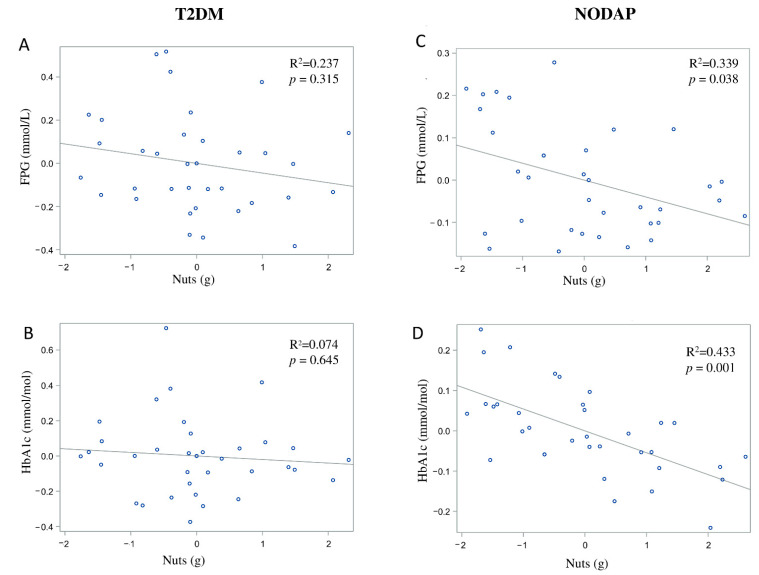
Associations between nuts intake and markers of glucose metabolism between T2DM (**A**,**B**) and NODAP group (**C**,**D**). Abbreviations: T2DM = Type 2 diabetes or prediabetes prior to acute pancreatitis. NODAP = New-onset diabetes or prediabetes after acute pancreatitis. HbA1c = glycated haemoglobin. FPG = fasting plasma glucose. R^2^ = adjusted R^2^ value (coefficient determinations). Footnotes: FPG, HbA1c, and nuts were log-trans. R^2^ values were adjusted for age, sex, BMI, energy intake (log-transformed), use of anti-diabetic medications, aetiology of pancreatitis, recurrence of pancreatitis, and presence of pancreatic necrosis.

In the NODAP group, intake of vegetables and nuts was significantly inversely associated with FPG in all the adjusted models (Table 2). Specifically, in the most adjusted model, every 1% of increase in intake of vegetables and nuts was associated with a 0.07% and a 0.04% decrease in FPG, correspondingly (Figure 4C and Figure 5C). Intake of neither fruit nor cereals was significantly associated with FPG in any model (Table 2). Intake of nuts was significantly inversely associated with HbA1c in both unadjusted and all adjusted models (Table 3). Specifically, in the most adjusted model, every 1% increase in intake of nuts was associated with a 0.05% decrease in HbA1c (Figure 5D). Intake of vegetables, fruit, and cereals was not significantly associated with HbA1c in any model (Table 3).

In the T2DM group, neither FPG nor HbA1c was significantly associated with any of the dietary sources of fibre in any model (Table 2 and Table 3).

In the NAP group, neither FPG nor HbA1c was significantly associated with any of the dietary sources of fibre in any model (Table 2 and Table 3).

## 4. Discussion

This was the first study to investigate the associations between dietary fibre intake and blood markers of glucose metabolism in individuals after AP. The included participants mainly had non-necrotising AP and none of them had extensive pancreatic necrosis on computed tomography. A key finding was that an increase in total fibre intake (after adjustment for age, sex, BMI, energy intake, use of anti-diabetic medications, and pancreatitis-related factors) was significantly associated with reduced FPG in the NODAP group, but not in the T2DM group. The subtypes of dietary fibre—insoluble and soluble fibre—were also significantly associated with reduced FPG in the NODAP group, but not the T2DM group (in the most adjusted model). Another key finding was that increased intake of dietary sources of fibre, specifically vegetables and nuts (but not cereals or fruit), was significantly associated with reduced FPG and HbA1c in the NODAP group in the most adjusted model. The above findings suggest that increased intake of dietary fibre may have a beneficial effect on glucose homeostasis in people with new-onset derangements of glucose metabolism after an attack of pancreatitis.

There are three possible mechanisms that may explain our findings. The first one relates to the physicochemical properties of dietary fibre. Soluble dietary fibre has a branched chemical structure or a polarised group, which allows it to have large water-binding capacity (including intra-dietary fibre particulate water and extra-dietary fibre particulate water) and swelling capacity [39]. Findings from a double-blind randomised controlled trial in healthy adults [40] showed that consumption of a carbohydrate meal (50 g of carbohydrate) with a high amount of soluble fibre (19.6 g of β-glucan) resulted in a lower postprandial plasma glucose level compared with a carbohydrate meal with a small amount of soluble fibre (4.5 g of β-glucan). Evidence from a randomised crossover controlled trial in individuals with Type 2 diabetes also showed that intake of liquid loaded with soluble fibre (7.5 g of oat β-glucan) was associated with a significant reduction in postprandial plasma glucose and insulin compared with intake of liquid without loading of soluble fibre [41]. Similarly, findings from the present study showed that intake of soluble fibre had an inverse association with FPG and explained 35.8% of variance in FPG levels. It is possible that ingestion of soluble dietary fibre leads to increased viscosity of the gut content [42], which results in a slower gastric emptying and a reduced absorption of carbohydrates from the gut [41,43].

Insoluble fibre is known to have lower water-binding capacity compared with soluble fibre. Unlike viscous soluble fibre, insoluble fibre is more likely to remain intact in the large intestine and is poorly fermented by the gut microbiota [42]. Earlier studies showed that insoluble fibre (e.g., cereals) was effective in stimulating the release of glucose-dependent insulinotropic polypeptide (GIP) from the intestinal K cells [44], which, in turn, improved insulin sensitivity. Our earlier study in individuals after an attack of AP found that GIP (but not the other incretin hormone—glucagon-like peptide-1) was significantly associated with insulin resistance in individuals with NODAP [27,45]. The present study showed that intake of insoluble fibre was inversely associated with FPG and explained 37.7% of variance in FPG levels. It is possible that an increased intake of insoluble dietary fibre results in an increased secretion of GIP in individuals with NODAP, leading to an increased secretion of insulin from β-cells [46].

The second possible mechanism is the interaction between dietary fibre and gut microbiota. Short-chain fatty acids (SCFAs) are produced by the gut microbiota in the large intestine by fermenting undigested dietary fibre [47]. Population-based studies showed that individuals with glucose intolerance had decreased production of SCFAs due to an altered gut microbiota profile [48,49]. Dietary fibre has been shown to be effective in stimulating the production of SCFAs, especially butyrate. Evidence showed that butyrate has a glucose lowering effect [50], which could be explained by its metabolic activity in the liver. After being produced in the large intestine, a fraction of butyrate is transported to the liver for ketone body synthesis. As a result, β-hydroxybutyrate is produced as one of the ketone bodies, which is then transported to peripheral tissues [51]. The 2021 CETUS randomised placebo-controlled trial in individuals with new-onset prediabetes after acute pancreatitis showed that mild exogenous ketosis results in a 14.5% reduction in plasma glucose levels and a significant increase in GIP level [52]. Based on the above, we hypothesise that there is a link between dietary fibre intake and circulating β-hydroxybutyrate levels. It is conceivable that increased intake of dietary fibre triggers an increase in butyrate production in the intestine, leading to increased levels of β-hydroxybutyrate. As a consequence, β-hydroxybutyrate may potentiate the insulinotropic effect of GIP and eventually result in reduced plasma glucose.

The third possible mechanism is the interaction between dietary fibre intake and the production of bile acids. Bile acids serve as ligands for farnesoid X receptor (FXR). Activation of FXR results in the expression of a fibroblast growth factor 15/19 (FGF15/19) in the intestine, which is associated with the reduction of plasma glucose levels by suppressing hepatic gluconeogenesis and inducing hepatic glycogen synthesis [53]. Increased intake of dietary fibre has been shown to stimulate the production of bile acids. Evidence from a randomised controlled crossover trial in healthy adults showed that the consumption of a diet rich in dietary sources of fibre at an average of 56 g/day (whole grain, vegetables, and legumes) resulted in an increased total bile acids synthesis [54]. Evidence from another randomised controlled trial showed that bile acid supplementation resulted in a significant increase in GIP [55]. Given that the present study showed a significant inverse association between dietary fibre and FPG in individuals with NODAP, it is possible that an increase in dietary fibre intake could lead to an increase in bile acid production, which activates the FXR pathway and increases GIP production. Since dietary fibre intake could affect the gut microbiota, SCFA, and bile acids production, the quantity and quality of dietary fibre intake may play an important role in glucose homeostasis in individuals after an attack of pancreatitis. Purposely-designed mechanistic studies are now warranted to elucidate the intricate relationship between dietary fibre, gut microbiota, and bile acids in this setting.

Since we observed the glucose-lowering effect of dietary fibre in the post-pancreatitis setting, it was important to consider the intake of common sources of dietary fibre. Higher nuts intake in the NODAP group was associated with significant reductions in both FPG and HbA1c. This finding in the post-pancreatitis setting was generally in line with earlier studies in other settings. A large-scale cross-sectional study in healthy adults demonstrated that consumption of tree nuts was associated with a significantly reduced risk of metabolic syndrome (including its component—elevated FPG) in people who had a high intake of nuts (above 18 g/day) [56]. A randomised controlled crossover trial in individuals with Type 2 diabetes showed a significant reduction in HbA1c (but not in FPG) in individuals after 24 weeks of daily almond intake (at 20% of energy intake) when compared to the control diet [57]. The significant association of both FPG and HbA1c with nuts intake in the present study suggests a possible long-term beneficial effect of nuts intake on glucose metabolism in post-pancreatitis individuals. Specifically, for every 1% increase of nuts intake, a reduction of 0.04% of FPG and 0.05% of HbA1c was observed in the NODAP group. A prospective longitudinal study is now warranted to investigate the effect of nuts intake, its frequency, and optimum daily intake on glucose metabolism in a post-pancreatitis setting. The other notable finding in the present study was the inverse association between vegetables intake and FPG in the NODAP group. Earlier studies showed an inverse association between the intake of vegetables and markers of glucose metabolism [17,18,19]. A meta-analysis of 10 prospective studies [18] showed that a higher intake of green leafy vegetables was associated with a reduced risk of Type 2 diabetes. Specifically, for every 0.2 servings/day increased intake of green leafy vegetables, there was a 13% lower risk of Type 2 diabetes. Evidence from a randomised controlled trial in people with Type 2 diabetes also showed that a diet high in vegetables (5–8 servings/day) resulted in a decrease in FPG when compared with the control diet (where no additional dietary targets were used) [19]. The present study found that, for every 1% increase of vegetable intake, FPG decreased by 0.07% in the NODAP group after accounting for several covariates. Based on the above, future nutritional guidelines for pancreatitis may need to recommend increased intake of vegetables and nuts in people after an attack of AP (provided that the dietary pattern is balanced).

There are several limitations of the present study. First, the ascertainment of dietary fibre intake was based on the self-reported FFQ, which means that a recall bias cannot be ruled out. However, the FFQ used in the present study was extensively validated [30], enabling the capture of a more accurate habitual dietary profile than other dietary measurement methods (e.g., 3-day food records) [58]. Second, for the purpose of the present study, dietary fibre was limited to non-starch polysaccharides only. Other sources of dietary fibre (e.g., resistant starch, oligosaccharides, and maltodextrins) were not captured in the used FFQ. However, non-starch polysaccharides are known to account for the majority of dietary fibre intake [31]. It is also important to acknowledge that the definition of insoluble and soluble fibre is principally based on their chemical properties instead of their physiological function. Currently, there is no accurate analytical method to distinguish soluble from insoluble fibre intake [59]. Third, sugar intake and protein intake may interact with dietary fibre and influence glucose homeostasis. The present study did not take into account all dietary covariates that are possibly associated with deranged blood glucose homeostasis. Given the relatively small sample size of the present study, inclusion of all possible dietary factors as covariates would have resulted in overfitting of our statistical models. Therefore, we elected to use energy intake as a single holistic factor covering most dietary variables in the statistical analyses. Larger studies in the future will be in a position to conduct a more granular analysis. Fourth, other chemical compounds in the food items that are rich in fibre may have affected the studied associations. For example, increased consumption of nuts is also associated with a higher intake of monounsaturated and polyunsaturated fatty acids, which may decrease FPG [60]. Intake of vegetables leads to a higher intake of antioxidants and flavonoids, which are known to improve insulin sensitivity [61]. Research into the above mentioned compounds in people with a history of AP is now warranted. Fifth, we were unable to calculate the glycaemic index, which is a metric for ascertaining the effect of a fixed amount of carbohydrate on the postprandial glycemic response [62]. Although earlier studies used the glycaemic index to predict the risk of chronic diseases, its use would not have been helpful in studying the associations in the present study. The concept of the glycaemic index includes a wide range of available carbohydrates in food but excludes other factors (e.g., antioxidants, vitamins, and minerals) affecting glycaemic control, which may confound the effect of dietary fibre [63]. The present study investigated dietary fibre alone, which provided specific guidance for future studies regarding the glucose-lowering effect of individual dietary factors in the post-pancreatitis setting. Sixth, the use of fibre supplements was not analyzed. Due to the limitation of the used software, we were unable to input food data from any supplements. Future studies should consider investigating all the possible sources of dietary fibre (i.e., both naturally occurring in food and present as processed fibre) in the post-pancreatitis setting. Seventh, although there is a body of evidence in the role of legume and soy intake in reducing the risk of diabetes [21,22,64], it was not analysed as an independent variable in the present study. Due to the limitations of the FETA software, a granular analysis of dietary fibre from different plant sources was not possible, resulting in the intake of legumes and other types of vegetables (such as leafy green vegetables and cruciferous vegetables) being classified as the intake of vegetables. Eighth, possible changes in dietary habits before and after an attack of AP were not investigated in the present study. However, given that the average time from the last attack of AP was 27 months and taking into account that the FFQ looked at the habitual dietary pattern over 12 months prior to the study visit, the data captured in the FFQ focused exclusively on the period after an attack of AP. Last, since the present study was a cross-sectional study, a causal relationship between dietary fibre intake and markers of blood glucose metabolism cannot be inferred. However, this was the first study investigating this relationship in the post-pancreatitis setting. Insights from the present study will be helpful in designing future prospective longitudinal studies of dietary intake in people after an attack of pancreatitis.

## 5. Conclusions

Habitual intake of dietary fibre (including both insoluble and soluble fibre) was significantly associated with glucose metabolism in the NODAP group. Given that intake of vegetables and nuts had significant inverse associations with FPG or HbA1c levels, increased consumption of these food items can be recommended to people after an attack of AP.

## Figures and Tables

**Table 1:** Characteristics of the study groups.

Characteristic	Overall	NODAP	T2DM	NAP	*p*
	(*n* = 108)	(*n* = 36)	(*n* = 36)	(*n* = 36)	
Age, years	58 (45–66)	60 (47–67)	60 (47–70)	51 (43–59)	0.201
Sex					0.421
Men	75	26	27	22	
Women	33	10	9	14	
Body Mass Index, kg/m^2^	27.4 (24.2–33.3)	27.5 (23.4–32.7)	28.8 (26.1–33.9)	26.7 (24.2–31.2)	0.391
Aetiology of pancreatitis					
Biliary	40	14	14	12	0.525
Alcohol-related	23	10	5	7	
Other	45	12	17	17	
Recurrence of pancreatitis					
Yes	31	14	8	9	0.383
No	77	22	28	27	
Pancreatic necrosis					0.235
Yes	6	4	1	1	
No	102	32	35	35	
Use of anti-diabetic medications					
Yes	5	0	5	0	**0.005**
No	103	36	31	36	
Energy, kcal	1797 (1230–2110)	1855 (1443–2167)	1705 (1285–2048)	1820 (1133–1996)	0.73
HbA1c, mmol/mol	40 (35–41)	39 (36–40)	47 (39–49)	34 (33–36)	**<0.001**
FPG, mmol/L	5.9 (5.0–6.4)	6.0 (5.1–6.7)	6.5 (5.5–6.9)	5.2 (4.8–5.5)	**0.002**

Abbreviations: NODAP = New-onset diabetes or prediabetes after acute pancreatitis. T2DM = Type 2 diabetes or prediabetes prior to acute pancreatitis. NAP = Normoglycaemia after acute pancreatitis. HbA1c = glycated haemoglobin. FPG = fasting plasma glucose. Footnotes: Data are presented as median (interquartile range) or frequency. Significance was set at *p* < 0.05. Significant values are shown in bold.

**Table 2:** Associations between dietary fibre intake and fasting plasma glucose.

	Model	NODAP (*n* = 36)				T2DM (*n* = 36)				NAP (*n* = 36)			
		*n*	B	*p*	Adjusted R^2^	*n*	B	*p*	Adjusted R^2^	*n*	B	*p*	Adjusted R^2^
Total fibre	1	36	−0.003	0.944	0.029	36	−0.096	0.207	0.018	36	0.021	0.594	0.02
	2	36	−0.064	0.218	0.119	36	−0.01	0.219	0.023	36	−0.015	0.694	0.127
	3	36	−0.129	**0.027**	0.208	36	−0.04	0.63	0.289	35	−0.086	0.292	0.069
	4	36	−0.154	**0.006**	0.398	36	−0.047	0.604	0.214	32	−0.145	0.153	0.108
Insoluble fibre	1	36	−0.016	0.745	0.026	36	−0.076	0.348	0.002	36	0.023	0.547	0.018
	2	36	−0.071	0.155	0.133	36	−0.074	0.375	0.001	36	−0.013	0.733	0.126
	3	36	−0.121	**0.026**	0.21	36	−0.025	0.759	0.286	35	−0.064	0.372	0.058
	4	36	−0.133	**0.01**	0.377	36	−0.033	0.715	0.21	32	−0.109	0.21	0.089
Soluble fibre	1	36	0.01	0.818	0.027	36	−0.088	0.207	0.018	36	0.001	0.981	0.03
	2	36	−0.034	0.455	0.092	36	−0.087	0.24	0.019	36	−0.02	0.542	0.133
	3	36	−0.109	0.051	0.18	36	−0.019	0.799	0.285	35	−0.081	0.194	0.087
	4	36	−0.13	**0.016**	0.358	36	−0.024	0.768	0.208	32	−0.12	**0.01**	0.136
Vegetables	1	36	−0.04	0.125	0.04	36	0.002	0.974	0.029	36	−0.009	0.801	0.028
	2	36	−0.065	**0.009**	0.253	36	0.006	0.91	0.024	36	−0.026	0.448	0.131
	3	36	−0.073	**0.004**	0.292	36	0.03	0.539	0.293	35	−0.027	0.487	0.033
	4	36	−0.069	**0.004**	0.41	36	0.027	0.615	0.214	32	−0.037	0.489	0.025
Fruit	1	36	0.002	0.917	0.029	36	−0.027	0.515	0.016	36	0.014	0.258	0.009
	2	36	−0.018	0.445	0.092	36	−0.028	0.524	0.011	36	0.008	0.545	0.133
	3	36	−0.025	0.286	0.102	36	−0.025	0.503	0.295	35	0.01	0.506	0.046
	4	36	−0.04	0.081	0.287	36	−0.023	0.562	0.216	32	0.003	0.861	0.025
Cereals	1	36	0.06	0.105	0.048	36	−0.069	0.284	0.005	36	0.014	0.577	0.019
	2	36	0.05	0.161	0.131	36	−0.063	0.341	0.004	36	0.007	0.745	0.126
	3	36	0.036	0.427	0.086	36	0.001	0.984	0.284	35	0.013	0.714	0.036
	4	36	0.034	0.386	0.206	36	−0.008	0.917	0.416	32	0.015	0.696	0.03
Nuts	1	36	−0.028	0.143	0.034	36	−0.027	0.517	0.016	36	−0.004	0.844	0.028
	2	36	−0.039	**0.032**	0.201	36	−0.035	0.44	0.005	36	−0.01	0.55	0.133
	3	36	−0.048	**0.01**	0.255	36	−0.034	0.371	0.304	35	−0.017	0.363	0.059
	4	36	−0.039	**0.038**	0.339	36	−0.045	0.315	0.237	32	−0.022	0.324	0.064

Abbreviations: NODAP = New onset diabetes or prediabetes after acute pancreatitis. T2DM = Type 2 diabetes or prediabetes prior to acute pancreatitis. NAP = Normoglycaemia after acute pancreatitis. B = β-coefficient. Adjusted R^2^ = adjusted coefficient of determinations. Footnotes: Model 1 was unadjusted. Model 2 was adjusted for age and sex. Model 3 was adjusted for age, sex, BMI, energy intake, and use of anti-diabetic medications. Model 4 was adjusted for age, sex, BMI, energy intake, use of anti-diabetic medications, aetiology of pancreatitis, recurrence of pancreatitis, and presence of pancreatic necrosis. Total fibre, insoluble fibre, soluble fibre, vegetables, fruit, cereals, nuts, and fasting plasma glucose were log-transformed. Data are presented as a β-coefficient, R^2^ value, and *p* value. Significance was set at *p* < 0.05. Significant values are shown in bold.

**Table 3:** Associations between dietary fibre intake and glycated haemoglobin.

	Model	NODAP (*n* = 36)				T2DM (*n* = 36)				NAP (*n* = 36)			
		*n*	B	*p*	Adjusted R^2^	*n*	B	*p*	Adjusted R^2^	*n*	B	*p*	Adjusted R^2^
Total fibre	1	36	−0.013	0.718	0.025	36	−0.052	0.45	0.012	36	0.059	**0.012**	0.151
	2	36	−0.057	0.143	0.141	36	−0.053	0.49	0.065	36	0.048	**0.033**	0.269
	3	36	−0.096	**0.033**	0.213	36	−0.012	0.885	0.079	34	0.052	0.277	0.277
	4	36	−0.082	0.093	0.154	35	0.018	0.842	0.067	31	−0.017	0.775	0.268
Insoluble fibre	1	36	−0.021	0.572	0.019	36	−0.035	0.621	0.021	36	0.057	**0.013**	0.148
	2	36	−0.054	0.163	0.135	36	−0.033	0.667	0.075	36	0.045	**0.042**	0.259
	3	36	−0.079	0.062	0.184	36	−0.001	0.982	0.079	34	0.042	0.319	0.272
	4	36	−0.067	0.135	0.135	35	0.002	0.973	0.065	31	−0.014	0.817	0.267
Soluble fibre	1	36	−0.004	0.895	0.028	36	−0.049	0.429	0.01	36	−0.004	0.895	0.028
	2	36	−0.042	0.227	0.121	36	−0.048	0.478	0.064	36	−0.042	0.227	0.122
	3	36	−0.093	**0.026**	0.223	36	−0.003	0.965	0.079	34	0.025	0.488	0.259
	4	36	−0.081	0.081	0.161	35	0.011	0.884	0.066	31	−0.028	0.501	0.28
Vegetables	1	36	−0.056	0.153	0.033	36	0.025	0.594	0.02	36	0.049	**0.029**	0.112
	2	36	−0.037	0.061	0.177	36	0.03	0.552	0.07	36	0.042	**0.042**	0.257
	3	36	−0.041	0.051	0.203	36	0.046	0.351	0.107	34	0.028	0.204	0.286
	4	36	−0.038	0.071	0.168	35	0.053	0.304	0.105	31	0.013	0.646	0.266
Fruit	1	36	−0.022	0.188	0.023	36	−0.016	0.667	0.023	36	0.015	0.064	0.073
	2	36	−0.03	0.086	0.162	36	−0.015	0.704	0.077	36	0.014	0.059	0.245
	3	36	−0.034	0.053	0.192	36	−0.015	0.689	0.084	34	0.009	0.257	0.28
	4	36	−0.029	0.131	0.137	35	−0.004	0.909	0.066	31	0.005	0.585	0.275
Cereals	1	36	0.041	0.146	0.034	36	−0.065	0.257	0.009	36	0.024	0.095	0.054
	2	36	0.036	0.182	0.131	36	−0.063	0.295	0.045	36	0.022	0.098	0.225
	3	36	0.038	0.261	0.12	36	−0.036	0.604	0.087	34	0.006	0.759	0.248
	4	36	0.035	0.311	0.095	35	−0.102	0.185	0.13	31	0.001	0.977	0.265
Nuts	1	36	−0.032	**0.022**	0.119	36	−0.021	0.585	0.02	36	0.003	0.789	0.028
	2	36	−0.038	**0.005**	0.286	36	−0.22	0.593	0.071	36	0.001	0.933	0.152
	3	36	−0.042	**0.002**	0.333	36	−0.024	0.546	0.09	34	−0.002	0.861	0.246
	4	36	−0.054	**0.001**	0.433	35	−0.02	0.645	0.074	31	−0.016	0.194	0.321

Abbreviations: NODAP = New onset diabetes or prediabetes after acute pancreatitis. T2DM = Type 2 diabetes or prediabetes prior to acute pancreatitis. NAP = Normoglycaemia after acute pancreatitis. B = β-coefficient. Adjusted R^2^ = adjusted coefficient of determinations. Footnotes: Model 1 was unadjusted. Model 2 was adjusted for age and sex. Model 3 was adjusted for age, sex, BMI, energy intake, and use of anti-diabetic medications. Model 4 was adjusted for age, sex, BMI, energy intake, use of anti-diabetic medications, aetiology of pancreatitis, recurrence of pancreatitis, and presence of pancreatic necrosis. Total fibre, insoluble fibre, soluble fibre, vegetables, fruit, cereals, nuts, and glycated haemoglobin were log-transformed. Data are presented as β-coefficient, R^2^ value, and *p* value. Significance was set at *p* < 0.05. Significant values are shown in bold.

## Data Availability

The data are not publicly available due to the ethical conduct in human research regulations.
